# T‐495, a novel low cooperative M_1_ receptor positive allosteric modulator, improves memory deficits associated with cholinergic dysfunction and is characterized by low gastrointestinal side effect risk

**DOI:** 10.1002/prp2.560

**Published:** 2020-01-28

**Authors:** Takao Mandai, Yuu Sako, Emi Kurimoto, Yuji Shimizu, Minoru Nakamura, Makoto Fushimi, Ryouta Maeda, Maki Miyamoto, Haruhide Kimura

**Affiliations:** ^1^ Neuroscience Drug Discovery Unit Research Takeda Pharmaceutical Company Limited Fujisawa Japan; ^2^ Biomolecular Research Laboratories Research Takeda Pharmaceutical Company Limited Fujisawa Japan; ^3^ Drug Metabolism and Pharmacokinetics Research Laboratories Research Takeda Pharmaceutical Company Limited Fujisawa Japan

**Keywords:** cooperativity, M_1_ muscarinic acetylcholine receptor, positive allosteric modulator, α‐synuclein

## Abstract

M_1_ muscarinic acetylcholine receptor (M_1_R) activation can be a new therapeutic approach for the treatment of cognitive deficits associated with cholinergic hypofunction. However, M_1_R activation causes gastrointestinal (GI) side effects in animals. We previously found that an M_1_R positive allosteric modulator (PAM) with lower cooperativity (α‐value) has a limited impact on ileum contraction and can produce a wider margin between cognitive improvement and GI side effects. In fact, TAK‐071, a novel M_1_R PAM with low cooperativity (α‐value of 199), improved scopolamine‐induced cognitive deficits with a wider margin against GI side effects than a high cooperative M_1_R PAM, T‐662 (α‐value of 1786), in rats. Here, we describe the pharmacological characteristics of a novel low cooperative M_1_R PAM T‐495 (α‐value of 170), using the clinically tested higher cooperative M_1_R PAM MK‐7622 (α‐value of 511) as a control. In rats, T‐495 caused diarrhea at a 100‐fold higher dose than that required for the improvement of scopolamine‐induced memory deficits. Contrastingly, MK‐7622 showed memory improvement and induction of diarrhea at an equal dose. Combination of T‐495, but not of MK‐7622, and donepezil at each sub‐effective dose improved scopolamine‐induced memory deficits. Additionally, in mice with reduced acetylcholine levels in the forebrain via overexpression of A53T α‐synuclein (ie, a mouse model of dementia with Lewy bodies and Parkinson's disease with dementia), T‐495, like donepezil, reversed the memory deficits in the contextual fear conditioning test and Y‐maze task. Thus, low cooperative M_1_R PAMs are promising agents for the treatment of memory deficits associated with cholinergic dysfunction.

**Abbreviations**
[^3^H]‐NMS: [^3^H]‐N‐methyl scopolamine
ACh: acetylcholine
AChEI: acetylcholinesterase inhibitor;
AD: Alzheimer's disease
BSA: bovine serum albumin
CaMKIIα: calcium/calmodulin‐dependent protein kinase II α
CFC: contextual fear conditioning
ChAT: choline acetyltransferase
CHO: Chinese hamster ovary
CS: conditioned stimulus
DLB: dementia with Lewy bodies
dTg: double transgenic
GAPDH: glyceraldehyde‐3‐phosphate dehydrogenase
GI: gastrointestinal
HBSS: Hanks’ Balanced Salt Solution
IP: inflection point
IP1: inositol 1‐phosphate
IP3: inositol 1,4,5‐trisphosphate
KO: knockout
Kp: brain‐to‐plasma concentration ratio
LC‐MS/MS: liquid chromatography‐tandem mass spectrometry
LiCl: lithium chloride
M_1_R: M_1_ muscarinic acetylcholine receptor
MK‐7622: 3‐((1*S*,2*S*)‐2‐hydroxycyclohexyl)‐6‐((6‐methylpyridin‐3‐yl)methyl)benzo[*h*]quinazolin‐4(3*H*)‐one
NDI: novelty discrimination index
NOR: novel object recognition
PAM: positive allosteric modulator
PDD: Parkinson's disease with dementia
PSD‐95: postsynaptic density‐95
SD: Sprague‐Dawley
sTg: single transgenic
T‐495: 8‐Chloro‐6‐((6‐chloropyridin‐3‐yl)methyl)‐3‐((1S,2S)‐2‐hydroxycyclopentyl)‐7‐methyl‐2,3‐dihydro‐4H‐1,3‐benzoxazin‐4‐one
tTA: tetracycline‐controlled transactivator
US: unconditioned stimulus
WT: wild‐type

## 1 INTRODUCTION

The neurotransmitter acetylcholine (ACh) is involved in synaptic plasticity and cognitive function.^1^, ^2^ Cholinergic neurotransmission dysfunction has been implicated in cognitive decline in various disorders, such as Alzheimer's disease (AD), dementia with Lewy bodies (DLB), and Parkinson's disease with dementia (PDD). The activity of choline acetyltransferase (ChAT), which is responsible for the synthesis of ACh, is severely reduced in the cortex and hippocampus of patients with AD, DLB, and PDD, although postsynaptic muscarinic ACh receptors are preserved.^3^, ^4^, ^5^, ^6^, ^7^ Furthermore, the reduction in ChAT activity correlates well with the dementia severity.^8^, ^9^, ^10^ Importantly, acetylcholinesterase inhibitors (AChEIs), such as donepezil and rivastigmine, have provided benefits to patients with AD, DLB, and PDD by increasing ACh levels in the synaptic cleft.^11^, ^12^ However, AChEIs have only modest efficacy and cause side effects, such as nausea and vomiting, leading to discontinuation of treatment.^13^, ^14^, ^15^ Thus, the development of novel therapies with higher efficacy and/or fewer side effects is needed for patients with AD, DLB, and PDD.

M_1_ muscarinic acetylcholine receptor (M_1_R) is highly expressed in the cerebral cortex and hippocampus, which are critical for cognitive function.^16^ Moreover, M_1_R deletion in mice led to cognitive impairment.^17^, ^18^ Furthermore, M_1_R activators reversed cognitive deficits in various animal models related to AD.^19^, ^20^, ^21^, ^22^ Importantly, xanomeline, an M_1_R/M_4_R agonist, produced robust improvement of the cognitive function in patients with AD, although the clinical development of this compound was discontinued due to severe cholinergic side effects, such as sweating and gastrointestinal (GI) dysfunction.^23^ Xanomeline‐induced cholinergic side effects occur likely due to its lack of selectivity and consequent activation of M_2_R and M_3_R in peripheral tissues. Therefore, selective M_1_R activation may provide a novel therapeutic strategy for cognitive impairment associated with cholinergic hypofunction.

However, surprisingly, recent studies have shown that even highly selective M_1_R activators retain the ability to produce cholinergic side effects such as diarrhea and vomiting in animals.^24^, ^25^, ^26^ Thus, identification of M_1_R positive allosteric modulators (PAMs) with better side effect profiles is essential for clinical application. We previously found that cooperativity (α‐value) is positively correlated with ileum contraction and that a low cooperative M_1_R PAM improves cognitive impairment without inducing diarrhea.^24^ These findings led to the hypothesis that fine adjustment of cooperativity of an M_1_R PAM is key to reducing the liability of GI side effects. Based on this hypothesis, we discovered a selective low cooperative M_1_R PAM, TAK‐071.^27^ TAK‐071 with a low α‐value of 199 exhibited a wider margin between cognitive improvement and diarrhea induction in rats compared with T‐662 as a reference M_1_R PAM with a high α‐value of 1786.^27^ Furthermore, combination of TAK‐071, but not T‐662, and an AChEI synergistically improved scopolamine‐induced cognitive impairment without exacerbating diarrhea.^27^ TAK‐071 is currently undergoing in clinical trials (ClinicalTrials.gov, Identifier: NCT02769065).

Recently, MK‐7622, a first‐in‐class high cooperative M_1_R PAM,^28^, ^29^ has shown no effect on cognition as adjunctive therapy with AChEIs in patients with AD.^30^ In the present study, to gain more insight into the differences between low and high cooperative M_1_R PAMs, we characterized the pharmacological profile of the novel low cooperative M_1_R PAM T‐495 using MK‐7622 as a control M_1_R PAM with a higher cooperative value. T‐495 exhibited a wider margin between memory improvement and induction of diarrhea than MK‐7622 in rats. Combination of T‐495, but not of MK‐7622, and donepezil at each subeffective dose ameliorated scopolamine‐induced memory impairment. In addition, in mice with reduced ACh levels in the forebrain due to A53T α‐synuclein overexpression (ie, a mouse model of DLB and PDD), T‐495 reversed memory deficits. These results suggest that cooperativity of M_1_R PAMs is an important parameter to obtain superior pharmacological profiles.

## 2 MATERIALS AND METHODS

### 2.1 Animals

All procedures involving animals were reviewed and approved by the Institutional Animal Care and Use Committee of Takeda Pharmaceutical Company Limited. All animals were maintained under a 12‐hour light/dark cycle, in a room with free access to food and water. Experiments were initiated after acclimation for at least 1 week.

Male ICR and C57BL/6J mice were supplied by CLEA Japan, Inc, and Sprague‐Dawley (SD) and Long‐Evans rats were purchased from Charles River Laboratories Japan, Inc and Japan SLC, Inc, respectively. C57BL/6‐*Chrm 1 ^tm1Stl^*/J wild‐type (WT) and homozygous knockout (M_1_R KO) mice were supplied by the Massachusetts Institute of Technology.

In this study, we used previously generated and characterized mice with overexpressed A53T α‐synuclein in the forebrain.^31^, ^32^ To obtain the mice, mice expressing A53T human α‐synuclein under the control of a tetO promoter on a B6C3F1 background (tetO‐A53T α‐synuclein mice) were purchased from The Jackson Laboratory (stock number 016976).^31^ In addition, mice expressing tetracycline‐controlled transactivator (tTA) under the control of the calcium/calmodulin‐dependent protein kinase II α (CaMKIIα) promoter on a C57BL/6J background (CaMKIIα‐tTA mice) were purchased from The Jackson Laboratory (stock number 007004) 33 and crossed to C3H/HeN mice (CLEA Japan, Inc). By crossing tetO‐A53T α‐synuclein mice and CaMKIIα‐tTA mice, nontransgenic, CaMKIIα‐tTA single transgenic (CaMKIIα‐tTA sTg), tetO‐A53T α‐synuclein single transgenic (A53T α‐syn sTg), and CaMKIIα‐tTA; tetO‐A53T α‐synuclein double transgenic (CaMKIIα‐tTA/A53T α‐syn dTg) mice were generated. To obtain the CaMKIIα‐tTA sTg and CaMKIIα‐tTA/A53T α‐syn dTg mice utilized in this study, CaMKIIα‐tTA sTg mice were further crossed with A53T α‐syn sTg mice. Male mice were used in all experiments. To shut off transgene expression during the embryonic and early postnatal period, pregnant and lactating mice were fed doxycycline‐containing chow (200 ppm, Japan SLC, Inc) from embryo transfer to the first 3 weeks after birth.

### 2.2 Chemical compounds

**Figure 1:**
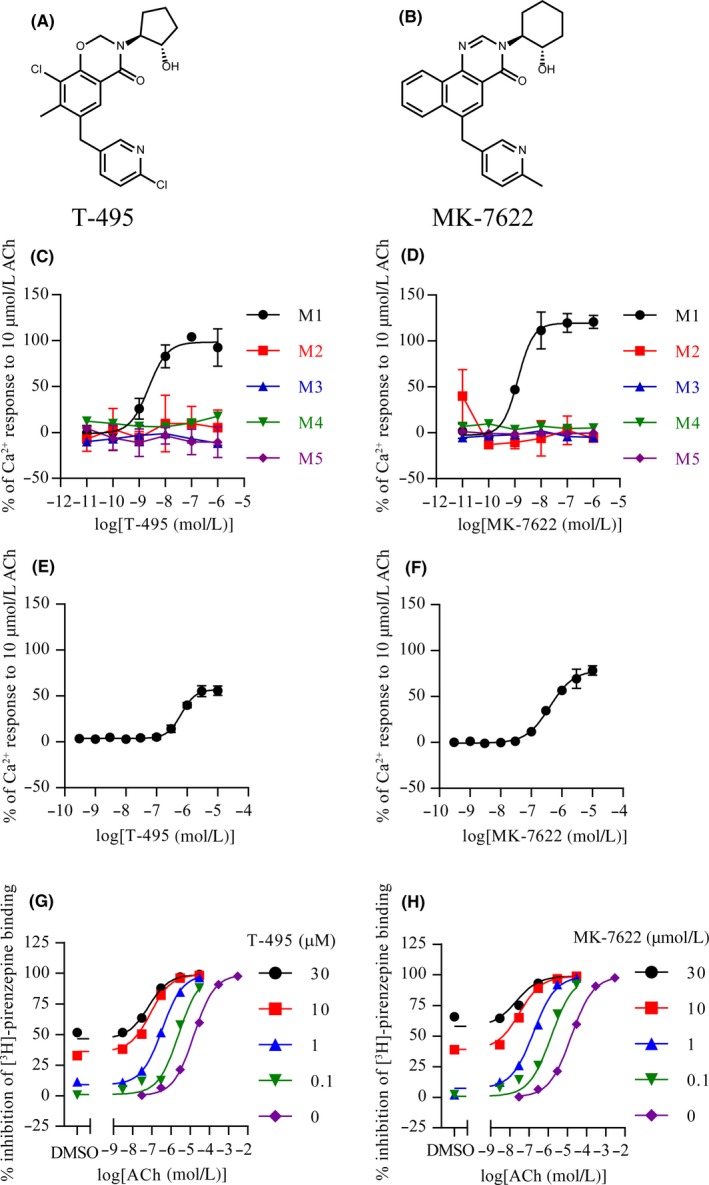
T‐495 and MK‐7622 selectively potentiate M_1_R with low and high cooperativity, respectively. (A and B) Chemical structures of T‐495 (A) and MK‐7622 (B). (C and D) Potentiation of ACh‐mediated Ca^2+^ mobilization by T‐495 (C) or MK‐7622 (D) in CHO‐K1 cells expressing human M_1_R‐M_5_R. The response to an EC_20_ concentration and 10 μmol/L of ACh was set as the 0% and 100% response, respectively. Data are presented as the mean ± SD (n = 3). (E and F) Effect of T‐495 (E) or MK‐7622 (F) on Ca^2+^ mobilization in the absence of ACh in CHO‐K1 cells expressing human M_1_R. The response to solvent and 10 μmol/L of ACh was set as the 0% and 100% response, respectively. Data are presented as the mean ± SD (n = 4). (G and H) Effect of T‐495 (G) or MK‐7622 (H) on [^3^H]‐pirenzepine binding in cell membranes from human M_1_R‐expressing cells (n = 2). Nonspecific binding was defined in the presence of 10 μmol/L atropine

T‐495 (Figure 1A: 8‐chloro‐6‐((6‐chloropyridin‐3‐yl)methyl)‐3‐((1*S*,2*S*)‐2‐hydroxycyclopentyl)‐7‐methyl‐2,3‐dihydro‐4*H*‐1,3‐benzoxazin‐4‐one) and MK‐7622 (Figure 1B: 3‐((1*S*,2*S*)‐2‐hydroxycyclohexyl)‐6‐((6‐methylpyridin‐3‐yl)methyl)benzo[*h*]quinazolin‐4(3*H*)‐one) were synthesized by Takeda Pharmaceutical Company Limited. T‐495 was synthesized according to the procedure described in the patent (WO2016208775, example 24).^34^ Donepezil hydrochloride was synthesized by Megafine Pharma Limited. Scopolamine hydrobromide and lithium chloride (LiCl) were purchased from Tocris Bioscience and Wako Pure Chemical Industries Limited, respectively. For in vitro experiments, T‐495 and MK‐7622 were dissolved in dimethyl sulfoxide (final concentration: 0.3% for Ca^2+^ mobilization assay and [^3^H]‐pirenzepine binding assay and 0.1% for spontaneous ileum contraction assay). For in vivo experiments, T‐495 and MK‐7622 were suspended in 0.5% methylcellulose in distilled water and administered orally (p.o.). Donepezil hydrochloride was dissolved in distilled water and administered orally. Scopolamine hydrobromide and LiCl were dissolved in saline and injected subcutaneously (s.c.). All compounds were dosed in a volume of 10 mL/kg body weight in mice and 2 mL/kg body weight in rats.

### 2.3 Ca^2+^ mobilization assay in cells expressing M_1_R–M_5_R

Chinese hamster ovary (CHO)‐K1 cells expressing M_1_R–M_5_R were plated on a 96‐well black, clear bottom plate at 30 000 cells/well and incubated at 37°C in an atmosphere of 5% CO_2_ for 1 day. On the day of the assay, cells were incubated with calcium dye buffer (Hanks’ Balanced Salt Solution (HBSS) containing 20 mmol/L HEPES, 0.1% fatty acid‐free bovine serum albumin (BSA), 0.08% pluronic F127 (Dojindo Laboratories), 2.5 μg/mL Fluo‐4 (Dojindo Laboratories), and 1.25 mmol/L probenecid (Dojindo Laboratories)) for 30 minutes at 37°C in an atmosphere of 5% CO_2_ and then incubated for 30 minutes at room temperature. To measure Ca^2+^ mobilization using CellLux (PerkinElmer), cells were stimulated with T‐495 or MK‐7622 (0.01‐1000 nmol/L for PAM activity; 0.3‐10 000 nmol/L for agonist activity) in assay buffer (HBSS containing 20 mmol/L HEPES and 0.1% fatty acid‐free BSA) with or without an EC_20_ concentration of ACh. The inflection point (IP) and EC_50_ values were calculated using the following equation by GraphPad Prism 5 software (GraphPad Software Inc):

$$Y=\text{Bottom}+\frac{\left(\text{Top}-\text{Bottom}\right)}{1+10^{\left(\left(\text{Log IP}\text{orEC}50-X\right)\times \text{HillSlope})\right)}}$$

where *X* and *Y* are the log concentration of a compound and the percentage of Ca^2+^ response, respectively, and Top and Bottom are the upper and lower plateaus, respectively.

### 2.4 [^3^H]‐pirenzepine binding assay

Cell membranes from FreeStyle 293 cells transiently expressing human M_1_R were incubated with T‐495 or MK‐7622 (0.1‐30 μmol/L), ACh (0.003‐3000 μmol/L), and 4 nmol/L [^3^H]‐pirenzepine (PerkinElmer) in assay buffer (20 mmol/L HEPES, 100 mmol/L NaCl, 10 mmol/L MgCl_2_, and 0.1% fatty acid free BSA) for 2 hours at room temperature. The binding was terminated by filtration through GF/C filter plates (PerkinElmer) using a cell harvester (PerkinElmer) and five washed with 300 μL of 50 mmol/L Tris‐HCl. The GF/C plates were dried at 42°C; then, 25 μL of microscint 0 (PerkinElmer) was added. Radioactivity was counted using Topcount (PerkinElmer). Nonspecific binding was defined in the presence of 10 μmol/L atropine. To calculate the cooperativity of a PAM, the [^3^H]‐pirenzepine binding assay data were fitted to the allosteric ternary complex model,^35^ using GraphPad Prism 5 software:

$$Y=\frac{\frac{\left[C\right]}{K_{C}}+\frac{\alpha_{\text{BC}}\left[B\right]\left[C\right]}{K_{B}K_{C}}}{1+\frac{\left[A\right]}{K_{A}}+\frac{\left[B\right]}{K_{B}}+\frac{\left[C\right]}{K_{C}}+\frac{\alpha_{\text{AB}}\left[A\right]\left[B\right]}{K_{A}K_{B}}+\frac{\alpha_{\text{BC}}\left[B\right]\left[C\right]}{K_{B}K_{C}}}$$

where *Y* is the fractional specific [^3^H]‐pirenzepine binding; [A], [B], and [C] are the concentrations of ACh, a PAM, and [^3^H]‐pirenzepine, respectively; *K*_A_, *K*_B_, and *K*_C_ are the equilibrium dissociation constants of ACh, a PAM, and [^3^H]‐pirenzepine, respectively; and α_AB_ and α_BC_ are the cooperativities between a PAM and ACh or [^3^H]‐pirenzepine, respectively.

### 2.5 [^3^H]‐N‐methyl scopolamine ([^3^H]‐NMS) binding assay

Cell membranes from FreeStyle 293 cells transiently expressing human M_1_R were incubated with T‐495 (0.01‐30 μmol/L) and 0.2 nmol/L [^3^H]‐NMS (PerkinElmer) in assay buffer as described above. After 2 hours at room temperature, the binding was terminated by filtration through GF/C filter plates using a cell harvester and washed five times with 300 μl of 50 mmol/L Tris‐HCl. GF/C plates were dried at 42°C and then 25 μl of microscint 0 was added. Radioactivity was measured by Topcount.

### 2.6 In vivo inositol 1‐phosphate (IP1) assay

Eight‐week‐old Long‐Evans rats, 8‐ to 9‐week‐old C57BL/6J mice, and 22‐ or 29‐week‐old M_1_R KO mice and their WT littermates were used in this study. On the day of the experiment, animals were placed in individual cages and acclimated for more than 1 hour. A test compound or vehicle was administered 2 hours before the animals were sacrificed. In the combination study, donepezil was administered 30 minutes after the administration of a test compound. LiCl (10 mmol/kg, s.c.) was injected 1 hour before sacrifice. In the repeated administration study, a test compound was administered to mice once daily for 13 days; on day 14, animals were treated with a test compound and LiCl (10 mmol/kg, s.c.) and then sacrificed. Animals were killed by decapitation, and blood was collected into tubes containing ethylenediaminetetraacetic acid for pharmacokinetic analysis; brains were quickly dissected, frozen on dry ice, and stored at −80°C until analysis. Brain tissues were homogenized in 19 (for rat tissues) or 39 (for mouse tissue) volumes of homogenization buffer (10 mmol/L HEPES pH 7.4, 50 mmol/L LiCl, 150 mmol/L NaCl, and 1% Triton X‐100), and the homogenate was incubated on a rotator for 1 hour, followed by centrifugation. The supernatant was diluted with 39 (for rat tissues) or 19 (for mouse tissues) volumes of dilution buffer (10 mmol/L HEPES pH 7.4, 50 mmol/L LiCl, and 150 mmol/L NaCl). IP1 and protein concentrations in the diluted supernatant were measured using IP‐One HTRF assay kit (Cisbio Bioassays) and BCA Protein Assay Kit (Thermo Fisher Scientific Inc), respectively, according to the manufacturers’ instructions. IP1 production was expressed as the ratio of IP1 to protein concentrations.

### 2.7 ACh quantification

Mice were sacrificed by focused microwave irradiation (MMW‐05, Muromachi Kikai Co., Ltd.). The frontal cortex and hippocampus were dissected and stored at −80°C until analysis. Tissues were homogenized with 39 volumes of ice‐cold methanol using a ShakeMaster Auto (BioMedical Science) at 1000 rpm for 2 minutes, followed by centrifugation at 20 000*g* for 5 minutes at 4°C. The supernatant (100 μL) was mixed with 10 μL of internal standard solution (ACh‐*d*_9_, Toronto Research Chemical) and 10 μL of distilled water. It was then centrifuged at 20 000 *g* for 5 minutes. Forty microliters of the supernatant was mixed with 60 μL of mobile phase A; subsequently, a 2 μL aliquot was analyzed by a liquid chromatography‐tandem mass spectrometry (LC‐MS/MS) system consisting of a Prominence 20A LC System (Shimadzu Co.) coupled to a 4000 QTRAP triple quadrupole‐mass spectrometer (AB Sciex, Framingham, MA). The chromatographic separation was performed using a LUNA C18(2) column (2 × 100 mm, 5 μm particles, Phenomenex) at 25°C. The mobile phase was composed of (A) 5 mmol/L heptafluorobutyric acid and 0.1% acetic acid in water and (B) 0.1% acetic acid in acetonitrile. The gradient was started and held at 1% (B) for 0.5 minutes, linearly increased to 100% (B) for over 4 minutes, and maintained at 100% (B) for 2 minutes, at a flow rate of 0.5 mL/minute.

The MS was operated in positive electrospray ionization mode with multiple reaction monitoring. The optimized source parameters for MS analysis were as follows: temperature, 400°C; curtain gas, 50 psi; collision gas, 10 psi; ion source gas 1, 50 psi; ion source gas 2, 50 psi; and ion spray voltage, 3000 V. The following transitions were monitored: *m*/*z* 146 → 87 for ACh and *m*/*z* 155 → 87 for ACh‐*d*_9_. The concentration of ACh was determined using a calibration curve constructed by plotting the peak area ratio of ACh to ACh‐*d*_9_ vs the nominal concentration of the analyte.

### 2.8 Automated capillary‐based western blot

Mice were sacrificed by decapitation, and their brains were quickly dissected, frozen on dry ice, and stored at −80°C until analysis. Tissues were homogenized and sonicated in 10 volumes of Cell Extraction Buffer (Thermo Fisher Scientific Inc) supplemented with complete Mini Protease Inhibitor Cocktail (Sigma‐Aldrich). The homogenates were incubated for 10 minutes on ice and centrifuged at 20 000 *g* for 15 minutes at 4°C. The supernatants were collected, and total protein concentrations were determined using BCA Protein Assay Kit (Thermo Fisher Scientific Inc). The expression level of target proteins was determined by capillary western blot (Wes, ProteinSimple), according to the manufacturer's instructions. Briefly, the supernatants were diluted with 0.1 × sample buffer to the appropriate concentration (800 μg/mL for the detection of drebrin, postsynaptic density‐95 (PSD‐95), M_1_R, and synaptophysin; 400 μg/mL for the detection of synapsin I). Additionally, four volumes of the diluted supernatants were mixed with one volume of 5 × fluorescent master mix and then incubated at 95°C for 5 minutes (except for the detection of M_1_R) or at 37°C for 60 minutes (for the detection of M_1_R). The following primary antibodies were used: mouse anti‐drebrin (1:50 dilution, catalog no. D029‐3, Medical & Biological Laboratories Co., Ltd.), rabbit anti‐PSD‐95 (1:50 dilution, catalog no. ab18258, Abcam plc), rabbit anti‐M_1_R (1:10 dilution, catalog no. mAChR‐M1‐Rb‐Af340, Frontier Institute Co. Ltd), rabbit anti‐synaptophysin (1:25 dilution, catalog no. ab32127, Abcam plc), rabbit anti‐synapsin I (1:50 dilution, catalog no. ab64581, Abcam plc), mouse anti‐glyceraldehyde‐3‐phosphate dehydrogenase (GAPDH, 1:100 dilution, catalog no. MAB374, Merck Millipore), and rabbit anti‐GAPDH (1:100 dilution, catalog no. 2118, Cell Signaling Technology, Inc). The prepared samples, antibody diluent 2, primary antibodies, anti‐rabbit or anti‐mouse secondary antibody, chemiluminescent substrate, and wash buffer were added to the appropriate wells of a prefilled microplate. Separation and detection were performed according to manufacturer's default settings. The peak area of the protein of interest was calculated using Compass software (ProteinSimple). The peak area of the target protein was normalized to that of GAPDH.

### 2.9 Behavioral testing

For all behavioral experiments, animals were acclimated to the experimental room for at least 1 hour before testing.

#### 2.9.1 Novel object recognition (NOR) test

On Day 1, 7‐week‐old Long‐Evans rats were individually placed into the test box fabricated from polyvinyl chloride (40‐cm square with 50‐cm high walls) without any objects for 10 minutes. On Day 2, a PAM and donepezil were administered 1 hour and 30 minutes prior to the acquisition trial, respectively; furthermore, scopolamine hydrobromide was injected 30 minutes prior to the acquisition trial. In the acquisition trial, each rat was placed in the test box containing two identical objects and allowed to explore them for 3 minutes. After an intertrial interval of 4 hours, each rat was again placed in the same test box containing one familiar and one novel objects and allowed to explore both for 3 minutes (retention trial). The behavior of each animal during the acquisition and retention trials was recorded on video, and the time spent exploring each object (licking, sniffing, or touching the object with the forepaws) was scored manually. Results were presented as novelty discrimination index (NDI) calculated as follows: the exploration time for the novel object/the exploration time for both objects × 100.

#### 2.9.2 Contextual fear conditioning (CFC) test

The CFC test was performed in a clear conditioning chamber (33 cm wide, 25 cm long, and 28 cm high; O’Hara & Co., Ltd.) surrounded by a sound‐attenuating box. The illumination in the chamber was maintained at 100 lux. Conditioned stimulus (CS) and unconditioned stimulus (US) were automatically delivered by a tone generator and a shock generator (O’Hara & Co., Ltd.), respectively. On Day 1 (habituation phase), vehicle was orally administered, and mice were placed in the conditioning chamber. They were allowed to freely explore for 120 seconds and then returned to their home cages. On Day 2 (conditioning phase), donepezil and T‐495 were orally administered to mice 2 and 1 hour, respectively, prior to conditioning. Mice were placed in the chamber for 120 seconds before the onset of a tone (CS, 60 dB for 30 s). The last 2 seconds of the CS were paired with a foot shock (US, 0.25 mA), and mice were removed and returned to their home cages 180 seconds after the US. On Day 3 (retention phase), donepezil and T‐495 were orally administered 2 and 1 hour, respectively, prior to the retention test. Mice were again placed in the same chamber for 180 seconds. Freezing behavior was analyzed using ImageJ FZ4 (O’Hara & Co., Ltd.) during the conditioning and retention phases.

#### 2.9.3 Y‐maze task

The Y‐maze was fabricated from gray plastic and consisted of three arms (40 cm long, 12 cm high, 3 cm wide at the bottom, and 10 cm wide at the top) with an angle of 120°. Visual cues were placed outside each arm, and the apparatus was illuminated at 10 lux. Two and 1 hour after oral administration of donepezil and T‐495, respectively, each mouse was placed at the end of one arm and allowed to freely explore the maze for 8 minutes. An arm entry was defined as all four paws of the mouse being in the arm, and the sequence of arm entries was monitored with a video camera and counted manually. An alternation was defined as successive entries into the three arms on overlapping triplet sets.^36^ The percentage of alternation was calculated as the ratio of actual to possible alternations (total number of arm entries minus 2) multiplied by 100.

### 2.10 Assessment of cholinergic side effects

Seven‐ or 8‐week‐old SD rats were individually placed into observation cages. After an acclimation period of at least 1 hour, a test compound was administered to the rats. In the combination study, donepezil was administered 30 minutes after the administration of a test compound. Cholinergic side effects, including diarrhea, lacrimation, salivation, miosis, and fasciculation, were assessed blindly, as described previously,^27^ and convulsion was scored using a modified Racine scale,^37^ as follows: stage 0, normal; stage 1, immobility; stage 2, forelimb and/or tail extension and rigid posture; stage 3, repetitive movements, head nodding, and gnawing; stage 4, rearing and falling; stage 5, continuous rearing and falling; stage 6, severe tonic‐clonic seizure with loss of postural control; and stage 7, death in the first 2 hours. The observations were carried out at 15 and 30 minutes and 1, 2, 4, 6, and 8 hours after the administration of T‐495 for the study of T‐495 alone, at 30 minutes and 1, 2, 4, and 6 hours after the administration of MK‐7622 for the study of MK‐7622 alone, and at 10 and 30 minutes and 1, 2, 4, and 6 hours after the administration of donepezil for the combination study.

### 2.11 Spontaneous ileum contraction

ICR mice were fasted overnight and sacrificed by decapitation, and the ileum was removed and the luminal contents were gently flushed out with ice‐cold modified Krebs buffer (NaCl, 120.7 mmol/L; KCl, 5.9 mmol/L; NaHCO_3_, 15.5 mmol/L; MgCl_2_, 1.2 mmol/L; NaH_2_PO_4_, 1.2 mmol/L; CaCl_2_, 2.5 mmol/L; glucose, 11.5 mmol/L). The ileum segment (approximately 5 mm in length) was suspended in a 10‐mL organ bath filled with oxygenated (95% O_2_ and 5% CO_2_) modified Krebs buffer at 37°C. One end of the ileum segment was tied to a hook and the other end was secured with a silk thread to an isometric force transducer (MLT050/A, ADInstruments) connected to a data acquisition system (PowerLab 8/30 ML870 and Octal Bridge Amp ML228, ADInstruments). The ileum segment was subjected to an initial tension of 0.3 to 0.4 g and was allowed to equilibrate for at least 30 minutes. After the equilibration period, spontaneous contractions were measured for 3 minutes (pretreatment) and then increasing concentrations of a test compound (0.01, 0.1, and 1 μmol/L) were cumulatively applied at 3‐minute intervals. LabChart software (ADInstruments) was used to analyze the spontaneous contractions. The mean amplitude of spontaneous contractions at each concentration was normalized to that observed at pretreatment.

### 2.12 Statistical analysis

Statistical analysis was performed using EXSUS (CAC EXICARE Corporation, Tokyo, Japan). The statistically significant differences between two groups were determined by Student's *t*‐test (for homogenous data) or Aspin‐Welch test (for nonhomogenous data), with significance set at *P* ≤ .05. For dose‐response studies, statistical comparison between vehicle‐ and drug‐treated groups was made by two‐tailed Williams’ test (for homogenous data) or Shirley‐Williams’ test (for nonhomogenous data), with *P* ≤ .05 considered a significant difference. Dunnett's test (for homogenous data) or Steel's test (for nonhomogenous data) was used to compare multiple independent groups, with significance set at *P* ≤ .05.

## 3 RESULTS

### 3.1 T‐495 and MK‐7622 were potent and selective M_1_R PAMs with different cooperativity

We first evaluated the PAM activities and selectivity of T‐495 (Figure 1A) and MK‐7622 (Figure 1B) by measuring Ca^2+^ influx in the presence of an EC_20_ concentration of ACh in CHO‐K1 cells expressing human M_1_R–M_5_R. T‐495 and MK‐7622 potentiated Ca^2+^ influx elicited by an EC_20_ concentration of ACh with IP values of 2.3 and 1.3 nmol/L, respectively, in CHO‐K1 cells expressing human M_1_R, whereas their IP values for human M_2_R–M_5_R were > 1,000 nmol/L (Table 1 and Figure 1C and D). Next, we measured Ca^2+^ influx in the absence of ACh to evaluate their agonist activities. T‐495 and MK‐7622 showed weak agonist activities with EC_50_ values of 649 and 407 nmol/L, respectively, in CHO‐K1 cells expressing human M_1_R (Table 1 and Figure 1E and F).

**Table 1:** Summary of PAM activity, agonist activity, and cooperativity of T‐495 and MK‐7622

	PAM activity		Agonist activity		Cooperativity	
	pIP	IP (nmol/L)	pEC_50_	EC_50_ (nmol/L)	Log α	α
T‐495	8.64 (8.34‐8.93)	2.3	6.19 (6.12‐6.25)	649	2.23 (2.07‐2.40)	170
MK‐7622	8.88 (8.72‐9.05)	1.3	6.39 (6.30‐6.49)	407	2.71 (2.48‐2.94)	511

Note

95% confidence intervals are shown in parentheses.

The selectivity of T‐495 at 10 μmol/L against a panel of 106 targets, including receptors, enzymes, ion channels, and transporters, was characterized (Eurofins Panlabs Taiwan Ltd., Taipei, Taiwan). No significant inhibition or stimulation (≥50%) was observed, except for two targets: 76% inhibition at the imidazoline I_2_ receptor and 58% inhibition at the dopamine transporter (Table S1). Furthermore, MK‐7622 was highly selective and exhibited inhibitory activities only against 5‐lipoxygenase and phosphodiesterase 4 in a selectivity panel.^28^

To evaluate the effects of T‐495 on the interaction between scopolamine and M_1_R, we conducted a [^3^H]‐NMS binding assay. T‐495 concentrations below 30 μmol/L did not inhibit [^3^H]‐NMS binding to M_1_R (Figure S1).

We previously identified cooperativity (α‐value) as a key parameter to reduce the risk of M_1_R PAMs‐induced GI side effects 24; thus, we assessed cooperativity of T‐495 and MK‐7622 using a [^3^H]‐pirenzepine binding assay. T‐495 and MK‐7622 caused a leftward shift in the displacement curve of [^3^H]‐pirenzepine binding by ACh (Figure 1G and H). The data of T‐495 and MK‐7622 were fitted to the allosteric ternary complex model, yielding α‐values of 170 and 511, respectively (Table 1). These results indicate that T‐495 is a potent and selective M_1_R PAM with low cooperativity, whereas MK‐7622, consistent with previous reports,^28^, ^29^ is a potent and selective M_1_R PAM with high cooperativity.

### 3.2 T‐495 and MK‐7622 increased IP1 production mediated by M_1_R activation in the rodent brain

**Figure 2:**
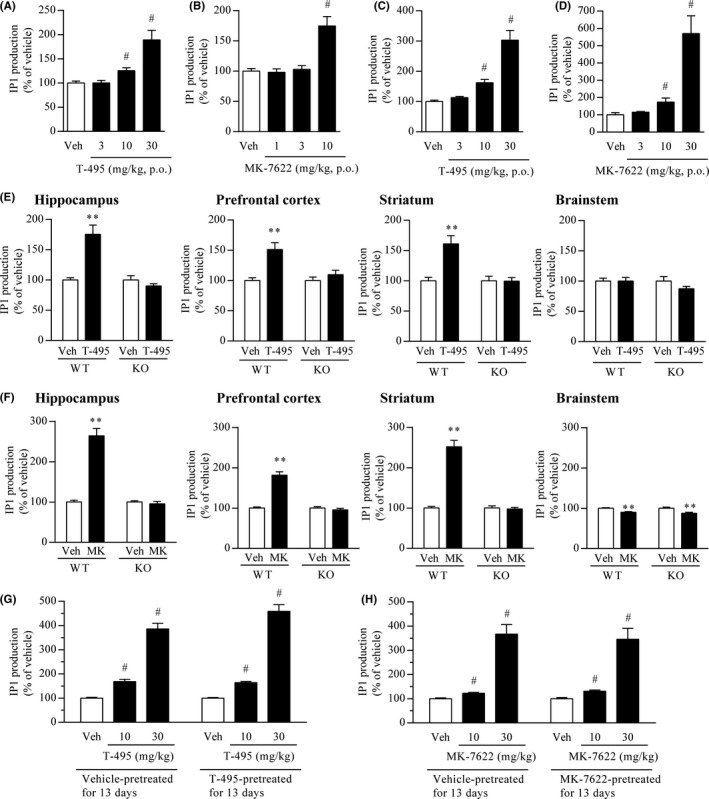
T‐495 and MK‐7622 increase IP1 production mediated by M_1_R activation in the rodent brain. (A and B) Effects of T‐495 (A) or MK‐7622 (B) on IP1 production in the rat hippocampus. One hour after oral administration of vehicle (Veh) or a test compound (T‐495:3, 10, and 30 mg/kg; MK‐7622:1, 3, and 10 mg/kg), animals were injected with LiCl (10 mmol/kg, s.c.). One hour after the LiCl injection, animals were sacrificed to collect the hippocampus. Concentrations of T‐495 and MK‐7622 in rat plasma and hippocampus are presented in Tables S2 and S3, respectively. Data are presented as the mean + SEM (n = 6). (C and D) Effects of T‐495 (C) or MK‐7622 (D) on IP1 production in the mouse hippocampus. One hour after oral administration of vehicle or a test compound (3, 10, and 30 mg/kg), animals were injected with LiCl (10 mmol/kg, s.c.). One hour after LiCl injection, animals were sacrificed to collect the hippocampus. Data are presented as the mean + SEM (n = 6). (E and F) Effects of T‐495 (E) or MK‐7622 (F) on IP1 production in the hippocampus, prefrontal cortex, striatum, and brainstem of wild‐type and M_1_R KO mice. One hour after oral administration of vehicle or a test compound (T‐495:10 mg/kg; MK‐7622 (MK): 20 mg/kg), mice were injected with LiCl (10 mmol/kg, s.c.). One hour after the LiCl injection, brain tissues were collected. Concentrations of T‐495 and MK‐7622 in the plasma and hippocampus of wild‐type and M_1_R KO mice are shown in Table S4. Data are presented as the mean + SEM (n = 10). (G and H) Effects of repeated treatment with T‐495 (G) or MK‐7622 (H) for 13 days on IP1 production in the mouse hippocampus. Vehicle or a test compound (10 mg/kg, p.o.) was administered to mice once daily for 13 days. On the 14th day, 1 hour after the administration of vehicle or a test compound (10 and 30 mg/kg, p.o.), mice were injected with LiCl (10 mmol/kg, s.c.). One hour after the LiCl injection, mice were sacrificed to collect the hippocampus. Basal IP1 levels in the mouse hippocampus after repeated treatment with vehicle or a test compound for 13 days are shown in Tables S5 (T‐495) and S6 (MK‐7622). Concentrations of T‐495 and MK‐7622 in the plasma and hippocampus of mice pretreated with vehicle or a test compound are shown in Tables S7 and S8, respectively. Data are presented as the mean + SEM (n = 10). **^#^** *P* ≤ .05 vs vehicle‐treated group by two‐tailed Shirley‐Williams’ test. **P ≤ .01 vs vehicle‐treated group by Student's *t*‐test or Aspin‐Welch *t*‐test

M_1_R activation leads to phospholipase C activation and subsequent inositol 1,4,5‐trisphosphate (IP3) generation. IP1, a downstream metabolite of IP3, is accumulated in the presence of LiCl, which inhibits IP1 degradation by suppressing inositol monophosphatase.^38^ Therefore, IP1 levels can be used to measure the activation levels of Gq protein‐coupled receptors, including M_1_R.^39^ To evaluate the in vivo activation of M_1_R by T‐495 and MK‐7622, IP1 levels after coadministration of T‐495 or MK‐7622 and LiCl were measured. In the rat hippocampus, T‐495 (10 and 30 mg/kg) and MK‐7622 (10 mg/kg) significantly increased IP1 production (Figure 2A and B). Under these experimental conditions, the brain‐to‐plasma concentration ratios (Kp) of T‐495 and MK‐7622 were 1.0‐1.4 and 0.1‐0.2, respectively (Tables S2 and S3). In the mouse hippocampus, T‐495 and MK‐7622 significantly increased IP1 production with a minimum effective dose of 10 mg/kg (Figure 2C and D).

To investigate whether the increases in IP1 production by T‐495 and MK‐7622 are mediated by M_1_R activation, we examined IP1 production in the mouse forebrain (hippocampus, prefrontal cortex, and striatum) and brainstem, where the expression levels of M_1_R are high and low, respectively.^16^ T‐495 at 10 mg/kg significantly increased IP1 production in the hippocampus, prefrontal cortex, and striatum, but not in the brainstem, of wild‐type mice (Figure 2E). MK‐7622 at 20 mg/kg increased and slightly reduced the IP1 production in the forebrain and brainstem of wild‐type mice (Figure 2F), respectively. In addition, T‐495 and MK‐7622 did not cause a significant increase in IP1 production in the hippocampus, prefrontal cortex, and striatum of M_1_R KO mice (Figure 2E and F). Concentrations of T‐495 and MK‐7622 in the plasma and brain of M_1_R KO mice were comparable to those observed in wild‐type mice (Table S4). These results indicate that T‐495 and MK‐7622 stimulate IP1 production through M_1_R activation. In contrast, MK‐7622 led to a slight reduction in IP1 production in the brainstem of both wild‐type and M_1_R KO mice, indicating that MK‐7622 may modulate molecular targets other than M_1_R.

M_1_R activation and signal transduction are strictly controlled by multiple molecular mechanisms such as the receptor internalization and downregulation.^40^ Persistent M_1_R activation by orthosteric agonists causes receptor internalization and downregulation in vitro.^41^, ^42^ To examine the effect of repeated administration of T‐495 or MK‐7622 on M_1_R signal transduction, we evaluated IP1 levels in the mouse hippocampus after 13 days of pretreatment with T‐495 at 10 mg/kg or MK‐7622 at 10 mg/kg. The magnitudes of the increase after pretreatment with T‐495 or MK‐7622 were comparable to those after single administration of T‐495 or MK‐7622 (Figure 2G and H). Basal IP1 levels and concentrations of T‐495 and MK‐7622 in the plasma and brain after 13 days of pretreatment were comparable with those after single administration (Tables S5–S8). These results suggest that repeated administration of T‐495 and MK‐7622 does not cause M_1_R desensitization.

### 3.3 T‐495 and MK‐7622 improved scopolamine‐induced memory impairment in a rat NOR test

**Figure 3:**
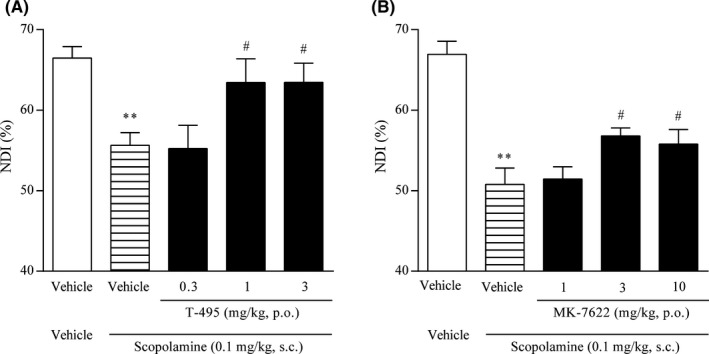
T‐495 and MK‐7622 improve scopolamine‐induced memory impairment in a rat NOR test. A test compound (T‐495 (A): 0.3, 1, and 3 mg/kg, p.o.; MK‐7622 (B): 1, 3, and 10 mg/kg, p.o.) and scopolamine (0.1 mg/kg, s.c.) were administered to rats 1 hour and 30 minutes prior to the acquisition trial, respectively. Data are presented as the mean + SEM (n = 6‐8). **P ≤ .01 vs vehicle‐vehicle‒treated group by Student's *t*‐test. **^#^** *P* ≤ .05 vs vehicle‐scopolamine‒treated group by two‐tailed Williams’ test

Scopolamine has been used to induce cholinergic dysfunction‐related cognitive deficits in healthy humans and animals.^43^ To evaluate the effects of T‐495 and MK‐7622 on object recognition memory, rats pretreated with scopolamine were used. In the retention trial, scopolamine significantly reduced NDI; T‐495 (1 and 3 mg/kg) and MK‐7622 (3 and 10 mg/kg) significantly reversed the scopolamine‐induced NDI reduction (Figure 3A and B). These data suggest that T‐495 and MK‐7622 have the potential to ameliorate cholinergic dysfunction‐related memory deficits.

### 3.4 T‐495 exhibited a wider margin than MK‐7622 between memory improvement and diarrhea induction in rats

**Figure 4:**
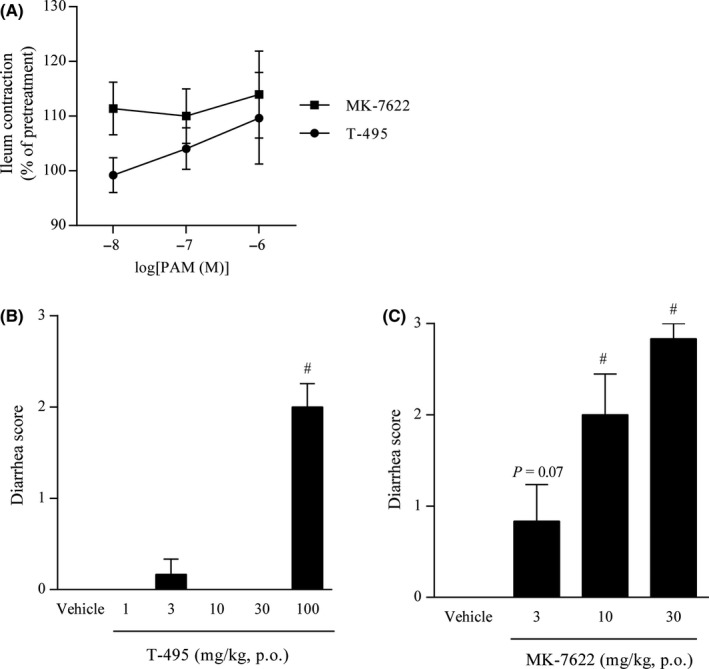
Effects of T‐495 and MK‐7622 on spontaneous ileum contraction and diarrhea score. (A) Effects of T‐495 and MK‐7622 on spontaneous ileum contraction. The mean amplitude of spontaneous contractions at each concentration was normalized to that at pretreatment. Data are presented as the mean ± SEM (n = 7‐10). (B and C) Diarrhea score. T‐495 (B; 1, 3, 10, 30, and 100 mg/kg, p.o.) or MK‐7622 (C; 3, 10, and 30 mg/kg, p.o.) was administered to rats, and the severity of diarrhea was scored. The highest score during the observation period was used for analysis. Data are presented as the mean + SEM (n = 6). **^#^** *P* ≤ .05 vs vehicle‐treated group by two‐tailed Williams’ test

We previously found that an M_1_R PAM with lower cooperativity had a lower impact on ileum contraction and a wider margin between memory improvement and diarrhea induction in rodents.^24^ To confirm these findings, the effects of T‐495 and MK‐7622 on ileum contraction were evaluated. T‐495 increased spontaneous ileum contraction at 1 μmol/L, whereas MK‐7622 increased that even at the lowest concentration tested (0.01 μmol/L; Figure 4A), suggesting that T‐495 has a lower impact on ileum motility.

Next, we characterized the cholinergic side effects induced by T‐495 (1‐100 mg/kg) and MK‐7622 (3‐30 mg/kg) in rats. T‐495 did not elicit any cholinergic side effects at doses up to 30 mg/kg (Table 2). However, T‐495 at 100 mg/kg significantly increased diarrhea score (Figure 4B) and incidence of diarrhea (Table 2) and caused convulsion and salivation in one of six rats (Table 2). MK‐7622 at 3 mg/kg tended to increase diarrhea score (*P* = .07; Figure 4C) and incidence of diarrhea (Table 3). Thus, T‐495 and MK‐7622 had 100‐fold and onefold dose differences, respectively, between memory improvement and induction of cholinergic side effects, including diarrhea, in rats.

**Table 2:** Observed side effects after oral administration of T‐495 in rats

Drug	Dose (mg/kg)	Loose or mucous stool or diarrhea	Convulsion	Lacrimation	Salivation	Miosis	Fasciculation
Vehicle	–	0/6	0/6	0/6	0/6	0/6	0/6
T‐495	1	0/6	0/6	0/6	0/6	0/6	0/6
T‐495	3	0/6	0/6	0/6	0/6	0/6	0/6
T‐495	10	0/6	0/6	0/6	0/6	0/6	0/6
T‐495	30	0/6	0/6	0/6	0/6	0/6	0/6
T‐495	100	5/6	1/6	0/6	1/6	0/6	0/6

Note

Observations were carried out at 15 and 30 minutes and 1, 2, 4, 6, and 8 hours after drug administration. The data are presented as the ratio of rats exhibiting side effects to the total number of rats (n = 6).

**Table 3:** Observed side effects after oral administration of MK‐7622 in rats

Drug	Dose (mg/kg)	Loose or mucous stool or diarrhea	Convulsion	Lacrimation	Salivation	Miosis	Fasciculation
Vehicle	‐	0/6	0/6	0/6	0/6	0/6	0/6
MK‐7622	3	2/6	0/6	0/6	0/6	0/6	0/6
MK‐7622	10	3/6	0/6	0/6	0/6	0/6	0/6
MK‐7622	30	6/6	0/6	0/6	0/6	0/6	0/6

Note

Observations were carried out at 30 minutes and 1, 2, 4, and 6 hours after drug administration. The data are presented as the ratio of rats exhibiting side effects to the total number of rats (n = 6).

### 3.5 Combining subeffective doses of T‐495, but not of MK‐7622, with donepezil improved scopolamine‐induced memory impairment without exacerbating cholinergic side effects in rats

Combination of an M_1_R PAM with an AChEI is expected to produce synergistic effects. In fact, additive or synergistic effects of an M_1_R PAM and an AChEI have been shown in behavioral paradigms and on M_1_R downstream signaling.^21^, ^27^, ^44^ Importantly, contrary to the low cooperative M_1_R PAM TAK‐071, the combination of T‐662, an M_1_R PAM with high cooperativity, and donepezil did not produce any additive or synergistic effects against scopolamine‐induced cognitive deficits.^27^

**Figure 5:**
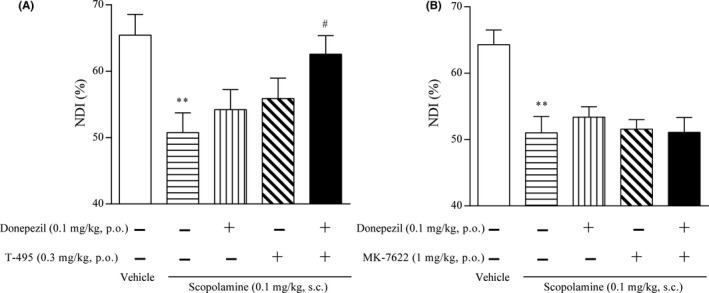
Effects of T‐495 or MK‐7622 in combination with donepezil on scopolamine‐induced memory impairment in a rat NOR test. A test compound (T‐495 at 0.3 mg/kg (A) or MK‐7622 at 1 mg/kg (B)), donepezil (0.1 mg/kg, p.o.), and scopolamine (0.1 mg/kg, s.c.) were administered 1, 0.5, and 0.5 hours, respectively, prior to the acquisition trial. Data are presented as the mean + SEM (n = 7‐8). **P ≤ .01 vs vehicle‐vehicle‐vehicle‒treated group by Student's *t*‐test. **^#^** *P* ≤ .05 vs vehicle‐vehicle‐scopolamine‒treated group by Dunnett's test

We evaluated the effects of combination of T‐495 or MK‐7622 and donepezil on scopolamine‐induced memory deficits in a rat NOR test. To avoid a ceiling effect, subeffective doses of T‐495 (0.3 mg/kg; Figure 3A), MK‐7622 (1 mg/kg; Figure 3B), and donepezil (0.1 mg/kg 27) were used in this study. In the retention trial, similar to TAK‐071,^27^ the combination of T‐495 and donepezil significantly improved scopolamine‐induced reduction in NDI (Figure 5A). When T‐495 (0.3 mg/kg) was combined with donepezil (0.1 mg/kg), no cholinergic side effects were observed (Table S9). In agreement with previous observations using T‐662, the combination of MK‐7622 and donepezil could not reverse scopolamine‐induced reduction in NDI (Figure 5B).

These results suggest that combination of T‐495, but not MK‐7622, and an AChEI have a potential to synergistically improve cholinergic dysfunction‐related memory deficits.

### 3.6 Validation of the CaMKIIα‐tTA/A53T α‐syn dTg mice as an animal model of DLB and PDD

Finally, to evaluate the efficacy of T‐495 on memory deficits in a disease‐relevant model, we used a recently reported mouse model of DLB and PDD (CaMKIIα‐tTA/A53T α‐syn dTg mice). Critical pathogenesis of DLB and PDD involves α‐synuclein accumulation. In the CaMKIIα‐tTA/A53T α‐syn dTg mice, A53T human α‐synuclein, which is an aggregation‐prone mutant, was specifically expressed in the forebrain, the region involved in cognitive functions, using the Tet‐off system and the CaMKIIα promoter.^31^ From 4 to 20 months of age, abnormal α‐synuclein accumulation was detected, which became progressively more profound.^32^ At 8 and 12 months of age, the mice exhibited cognitive deficits in the CFC test.^32^ Thus, in the current study, 12‐month‐old mice were used, and age‐matched CaMKIIα‐tTA sTg mice were utilized as a control for CaMKIIα‐tTA/A53T α‐syn dTg mice.

#### 3.6.1 ACh content and synaptic protein levels in the frontal cortex and hippocampus of the CaMKIIα‐tTA/A53T α‐syn dTg mice

**Figure 6:**
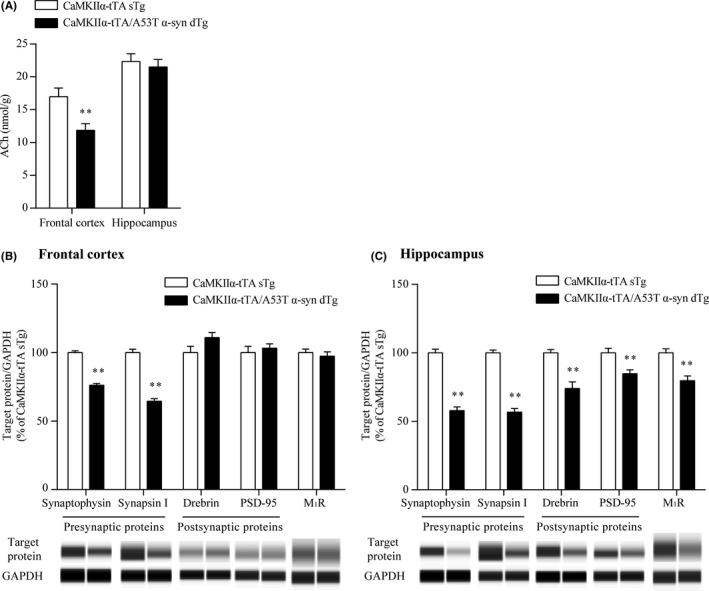
ACh content and synaptic protein and M_1_R levels in the frontal cortex and hippocampus of the CaMKIIα‐tTA/A53T α‐syn dTg mice. The frontal cortex and hippocampus were dissected from 12‐month‐old mice. (A) The ACh content in the tissues was measured by LC‐MS/MS. Data are presented as the mean + SEM (n = 21‐24), and statistical significance of the differences between the CaMKIIα‐tTA sTg and CaMKIIα‐tTA/A53T α‐syn dTg mice was determined using Student's *t*‐test (**P ≤ .01). (B and C) Presynaptic and postsynaptic proteins in the frontal cortical (B) and hippocampal (C) lysates from the CaMKIIα‐tTA sTg and CaMKIIα‐tTA/A53T α‐syn dTg mice were determined by an automated capillary western blot system. Synaptic protein levels were normalized to the levels of GAPDH, which was a loading control. Results are expressed as percentages of the values obtained from age‐matched CaMKIIα‐tTA sTg mice and are presented as the mean + SEM (n = 10). The statistical significance of the difference between the CaMKIIα‐tTA sTg and CaMKIIα‐tTA/A53T α‐syn dTg mice was determined using Student's *t*‐test or Aspin‐Welch test (**P ≤ .01). Representative images of capillary western blot are shown below the quantified results in panels B and C

To investigate the impact of abnormal α‐synuclein accumulation on the cholinergic system, we evaluated ACh levels in the frontal cortex and hippocampus, where marked reduction in ChAT activity has been observed in patients with DLB and PDD.^4^, ^5^, ^7^ ACh levels in the frontal cortex, but not in the hippocampus, of the CaMKIIα‐tTA/A53T α‐syn dTg mice were significantly lower than those of the CaMKIIα‐tTA sTg mice (Figure 6A).

In the brains of patients with DLB and PDD, most α‐synuclein aggregates are located in the presynaptic terminals and cause synaptic dysfunction via significant reduction of pre‐ and postsynaptic proteins, such as synaptophysin and drebrin.^45^, ^46^, ^47^ To quantitatively evaluate the effects of abnormal α‐synuclein accumulation on synaptic proteins in the frontal cortex and hippocampus, we assessed synaptophysin and synapsin I levels as presynaptic markers and drebrin and PSD‐95 levels as postsynaptic markers using capillary western blot. In the frontal cortex of the CaMKIIα‐tTA/A53T α‐syn dTg mice, synaptophysin and synapsin I levels were significantly reduced by 24% and 35%, respectively, whereas no significant drebrin and PSD‐95 reduction was observed compared to the CaMKIIα‐tTA sTg mice (Figure 6B). In the hippocampus of the CaMKIIα‐tTA/A53T α‐syn dTg mice, 42% and 43% reduction in synaptophysin and synapsin I, respectively, and 26% and 15% reduction in drebrin and PSD‐95, respectively, were observed (Figure 6C). We also evaluated the expression levels of M_1_R in the CaMKIIα‐tTA/A53T α‐syn dTg mice. Although the M_1_R levels were unaltered in the frontal cortex (Figure 6B), a 20% reduction in M_1_R levels was observed in the hippocampus of the CaMKIIα‐tTA/A53T α‐syn dTg mice (Figure 6C).

#### 3.6.2 Memory deficits in the CaMKIIα‐tTA/A53T α‐syn dTg mice and efficacy of donepezil

**Figure 7:**
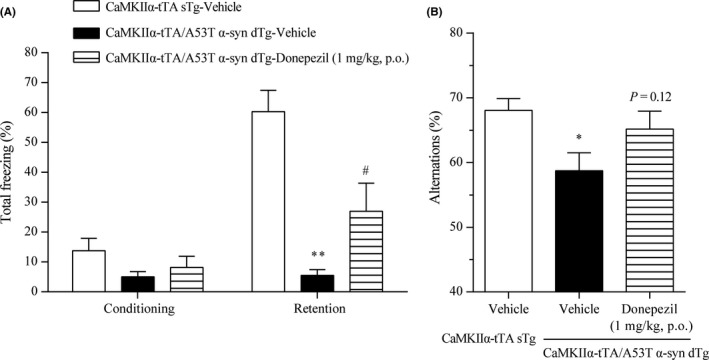
Effects of donepezil on the memory deficits in the CaMKIIα‐tTA/A53T α‐syn dTg mice. (A) The CFC test was performed to evaluate associative learning of 12‐month‐old CaMKIIα‐tTA sTg and CaMKIIα‐tTA/A53T dTg mice. The percentage of freezing behavior was analyzed during the conditioning and retention phases. Vehicle or donepezil (1 mg/kg) was orally administered 2 hours prior to both the conditioning and retention phases. (B) The Y‐maze task was performed to evaluate spatial working memory of 12‐month‐old CaMKIIα‐tTA sTg and CaMKIIα‐tTA/A53T dTg mice. The percentage of alternations was measured. Vehicle or donepezil (1 mg/kg) was orally administered 2 hours prior to the test. Data are presented as the mean + SEM (n = 10); statistical significance between the vehicle‐treated CaMKIIα‐tTA sTg and CaMKIIα‐tTA/A53T α‐syn dTg mice was determined using Student's *t*‐test or Aspin‐Welch test (*P ≤ .05; **P ≤ .01). Significant differences between vehicle‐ and donepezil‐treated CaMKIIα‐tTA/A53T α‐syn dTg mice were determined by Student's *t*‐test or Aspin‐Welch test (^#^ *P* ≤ .05)

Next, we assessed the memory function of the CaMKIIα‐tTA/A53T α‐syn dTg mice. First, the CFC test was used to measure associative learning in the CaMKIIα‐tTA/A53T α‐syn dTg mice. No significant difference in the level of freezing behavior during the conditioning phase between the CaMKIIα‐tTA sTg and CaMKIIα‐tTA/A53T α‐syn dTg mice was observed (Figure 7A). In contrast, consistent with a previous report,^32^ the CaMKIIα‐tTA/A53T α‐syn dTg mice exhibited a significantly reduced level of freezing behavior during the retention phase compared to the CaMKIIα‐tTA sTg mice (Figure 7A); these results suggest that associative learning is impaired in the CaMKIIα‐tTA/A53T α‐syn dTg mice. Cognitive deficits observed in patients with DLB and PDD include impairment of working memory.^48^, ^49^, ^50^ Thus, we also evaluated spatial working memory in CaMKIIα‐tTA/A53T α‐syn dTg mice using the Y‐maze task. The percentage of alternations in the CaMKIIα‐tTA/A53T α‐syn dTg mice was significantly reduced compared to that in the CaMKIIα‐tTA sTg mice (Figure 7B), suggesting that working memory was impaired in the CaMKIIα‐tTA/A53T α‐syn dTg mice.

We then evaluated whether donepezil, which improves cognitive decline in patients with DLB and PDD,^51^, ^52^ can reverse the memory deficits observed in the CaMKIIα‐tTA/A53T α‐syn dTg mice. Donepezil at 1 mg/kg improves memory deficits in a mouse model of AD 53, 54; thus, a dose of 1 mg/kg was used in this study. In the CFC test, donepezil treatment (1 mg/kg) before both the conditioning and retention phases significantly reversed the reduced level of freezing behavior during the retention phase in the CaMKIIα‐tTA/A53T α‐syn dTg mice; however, donepezil did not affect the level of freezing behavior during the conditioning phase (Figure 7A). Furthermore, in the Y‐maze task, donepezil treatment (1 mg/kg) tended to reverse the reduced percentage of alternations in the CaMKIIα‐tTA/A53T α‐syn dTg mice (*P* = .12; Figure 7B).

Together with the results of a previous study,^32^ our data imply that CaMKIIα‐tTA/A53T α‐syn dTg mice, the recently reported mouse model of DLB and PDD, replicate multiple key features of DLB and PDD. Therefore, CaMKIIα‐tTA/A53T α‐syn dTg mice are a good animal model for DLB and PDD.

### 3.7 T‐495 reversed the memory impairment observed in the CaMKIIα‐tTA/A53T α‐syn dTg mice

We evaluated the effects of T‐495 on memory deficits in CaMKIIα‐tTA/A53T α‐syn dTg mice. T‐495 at 1 and 3 mg/kg significantly improved scopolamine‐induced memory impairment in a rat NOR test (Figure 3). Furthermore, hippocampal IP1 production was significantly increased at the same dose in rats and mice (Figure 2A and B). Thus, we decided to use a dose of 3 mg/kg.

**Figure 8:**
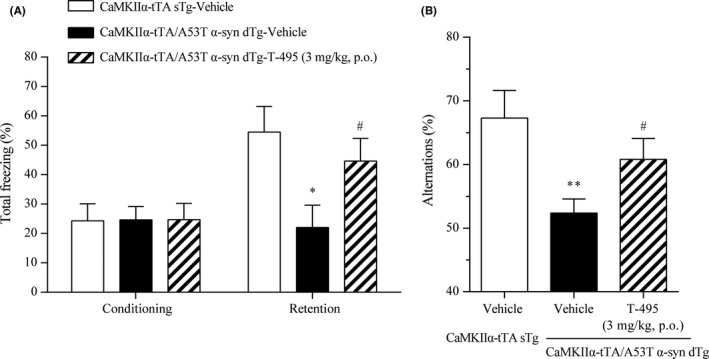
Effects of T‐495 on the memory deficits in the CaMKIIα‐tTA/A53T α‐syn dTg mice. (A) The CFC test was performed at 12 months of age. The percentage of freezing behavior was analyzed during the conditioning and retention phases. Vehicle or T‐495 (3 mg/kg) was administered orally 1 hour prior to both the conditioning and retention phases. (B) The Y‐maze task was performed at 12 months of age. The percentage of alternations was measured. Vehicle or T‐495 (3 mg/kg) was orally administered 1 hour prior to the test. Data are presented as the mean + SEM (n = 10‐15), and statistical significance between the vehicle‐treated CaMKIIα‐tTA sTg and CaMKIIα‐tTA/A53T α‐syn dTg mice was determined using Student's *t*‐test or Aspin‐Welch test (*P ≤ .05; **P ≤ .01). Significant differences between vehicle‐ and T‐495‐treated CaMKIIα‐tTA/A53T α‐syn dTg mice were determined by Student's *t*‐test or Aspin‐Welch test (^#^ *P* ≤ .05)

In this cohort, the level of freezing behavior during the retention phase in the CFC test and the percentage of alternations in the Y‐maze task were significantly reduced in the CaMKIIα‐tTA/A53T α‐syn dTg mice compared to the CaMKIIα‐tTA sTg mice (Figure 8A and B). In the CFC test, T‐495 treatment (3 mg/kg) before both conditioning and retention phases significantly reversed the reduced level of freezing behavior during the retention phase in the CaMKIIα‐tTA/A53T α‐syn dTg mice; however, T‐495 did not change the level of freezing behavior during the conditioning phase (Figure 8A). Moreover, in the Y‐maze task, T‐495 (3 mg/kg) significantly reversed the reduced percentage of alternations in the CaMKIIα‐tTA/A53T α‐syn dTg mice (Figure 8B).

## 4 DISCUSSION

M_1_R activation may be a promising therapeutic approach to improve cognitive impairment associated with cholinergic dysfunction.^55^ However, highly selective M_1_R PAMs have been reported to induce cholinergic side effects, particularly GI side effects such as diarrhea, in animals.^24^, ^25^, ^26^ In fact, in a recently published phase 2 clinical trial of MK‐7622 in patients with AD, diarrhea was the most common adverse effect associated with MK‐7622 treatment at the estimated effective dose, affecting 15% of the patients.^30^ Therefore, the identification of M_1_R PAMs with minimal GI side effects is needed to improve the clinical utility of this drug class.

We have previously determined that fine‐tuning of cooperativity is essential to identify M_1_R PAMs with a wider safety margin.^24^ Using this approach, we identified the low cooperative M_1_R PAM TAK‐071 (α‐value of 199).^27^ In rats, TAK‐071 exhibited a 33‐fold difference between doses improving cognitive function and doses inducing diarrhea, whereas the high cooperative M_1_R PAM T‐662 (α‐value of 1786) showed no dose difference between the two effects.^27^ Furthermore, interestingly, combination of subeffective doses of TAK‐071, but not T‐662, and an AChEI currently used for the treatment of AD synergistically improved scopolamine‐induced cognitive deficits detected in the NOR test.^27^ Here, using the novel low cooperative M_1_R PAM T‐495 (α‐value of 170) and the clinically tested high cooperative M_1_R PAM MK‐7622 (α‐value of 511) (Figure 1), we further investigated the pharmacological differences between low and high cooperative M_1_R PAMs. Consistent with previous observations using TAK‐071 and T‐662, T‐495 elicited cholinergic side effects, including diarrhea, at a 100‐fold higher dose than that required for the improvement of scopolamine‐induced memory deficits in the NOR test (Table 2 and Figures 3A and 4B), whereas MK‐7622 showed memory improvement and diarrhea induction at an equal dose (Table 3 and Figures 3B and 4C). Furthermore, potential efficacy was observed with the combination of T‐495, but not of MK‐7622, and donepezil when a subeffective dose of each drug was used (Figure 5A and B); however, combination studies using multiple doses of each drug could not be conducted owing to a potential “ceiling effect” in memory enhancement. Notably, MK‐7622 did not improve cognition as adjunctive therapy with AChEIs in patients with AD.^30^ ACh release is spatiotemporally controlled in tissues, and an appropriate coordinated cholinergic system activation in the brain would be required for cognitive performance.^56^, ^57^ Unlike M_1_R agonists, M_1_R PAMs are expected to boost the action of ACh and maintain its spatiotemporal characteristics. However, high cooperative M_1_R PAMs may disrupt the spatiotemporally controlled manner of M_1_R activation via a robust increase in the binding affinity of ACh to M_1_R, especially when the ACh levels are high by coadministration of an AChEI.

Recent studies have implicated the association of M_1_R PAM agonism with cholinergic side effects.^58^, ^59^ Both T‐495 and MK‐7622 showed agonist activity in CHO‐K1 cells expressing human M_1_R. The margins between PAM and agonist activities of T‐495 (282‐fold) and MK‐7622 (313‐fold) were comparable (Figure 1C–F). Therefore, cooperativity, but not agonist activity, of M_1_R PAMs may contribute to the improved and superior pharmacological profile. However, to more precisely quantify agonist activity, physiological receptors in brain slices and primary cells, rather than cell lines, should be used, because agonist activity is greatly affected by various factors including the degree of receptor reserve.^60^ Unfortunately, only low‐throughout electrophysiological assays using brain slices are available for characterization of the agonist activity of M_1_R PAMs. Further efforts to establish higher throughput assays for the discovery of a variety of potent M_1_R PAMs with a wider margin between PAM activity and agonist activity would be needed.

To evaluate the procognitive efficacy of T‐495 and MK‐7622, scopolamine‐induced memory deficits in a NOR test were used. Both compounds have a potential to improve scopolamine‐induced memory deficits by directly inhibiting scopolamine binding to M_1_R. We previously showed that other low and high cooperative M_1_R PAMs, TAK‐071 and T‐662, did not inhibit the binding of scopolamine to M_1_R at concentrations required for their procognitive efficacy.^27^ In this study, we observed that T‐495 did not inhibit [^3^H]‐NMS binding to M_1_R at up to 100‐fold higher concentration than that required for the procognitive efficacy (Table S2 and Figure S1). Thus, the inhibition of binding between scopolamine and M_1_R by M_1_R PAMs may not contribute to their memory enhancing effects in the rat NOR test.

To model α‐synuclein‐associated diseases, including DLB and PDD, multiple transgenic animals overexpressing wild‐type or mutant α‐synuclein under the control of various promoters have been generated.^61^ However, in these mice, α‐synuclein is broadly expressed in central nervous system neurons, including regions not affected in DLB and PDD. Recently, an interesting novel transgenic mouse associated with DLB and PDD (CaMKIIα‐tTA/A53T α‐syn dTg mice) has been generated using the Tet‐off system and the CaMKIIα promoter.^31^, ^32^ CaMKIIα‐tTA/A53T α‐syn dTg mice express A53T human α‐synuclein in the neurons of the forebrain, the region involved in cognitive function, and exhibit cognitive deficits accompanied by α‐synuclein pathology.^31^, ^32^ To determine whether the CaMKIIα‐tTA/A53T α‐syn dTg mice are a valid animal model of DLB and PDD, we further characterized their behavioral, molecular, and pharmacological phenotypes. CaMKIIα‐tTA/A53T α‐syn dTg mice replicate the following key features of DLB and PDD: (1) α‐synuclein pathology 32; (2) cholinergic deficit in the cerebral cortex (Figure 6A); (3) reduction in pre‐ and postsynaptic proteins (Figure 6B and C); (4) cerebral and hippocampal atrophy 32; (5) memory deficits (Figure 7)^32^; and (6) amelioration of memory deficits by donepezil (Figure 7). Thus, the CaMKIIα‐tTA/A53T α‐syn dTg mice are a good animal model for DLB and PDD and may assist in elucidating the molecular mechanisms underlying DLB and PDD and testing new therapeutic agents.

Cognitive deficits observed in the CaMKIIα‐tTA/A53T α‐syn dTg mice were correlated with α‐synuclein pathology in the hippocampus 32; however, the underlying molecular mechanism remains unknown. In the hippocampus of the CaMKIIα‐tTA/A53T α‐syn dTg mice, the expression levels of M_1_R and other postsynaptic proteins, such as drebrin and PSD‐95, were reduced (Figure 6C). In M_1_R KO mice, cognitive deficits were observed.^17^, ^18^ Thus, reduced M_1_R signaling in the hippocampus may contribute to memory deficits in the CaMKIIα‐tTA/A53T α‐syn dTg mice. T‐495 treatment may improve memory deficits via restoration of the reduced M_1_R signaling in the CaMKIIα‐tTA/A53T α‐syn dTg mice (Figure 8), but further studies are required to confirm this.

In summary, together with our previous findings,^27^ the present data demonstrate that low and high cooperative M_1_R PAMs exhibit different pharmacological profiles with regards to a safety margin between memory improvement and cholinergic side effects and combination efficacy with AChEIs. Therefore, fine‐tuning of M_1_R PAM cooperativity is key to achieving superior pharmacological profiles. Low cooperative M_1_R PAMs, such as TAK‐071 and T‐495, may represent a novel therapeutic agent for memory deficits associated with cholinergic hypofunction.

## DISCLOSURE

The authors declare no other conflict of interest.

## AUTHORS CONTRIBUTIONS

Mandai, Sako, Kurimoto, Shimizu, Fushimi, Maeda, Miyamoto, and Kimura *participated in research design.* Mandai, Sako, Kurimoto, Shimizu, Maeda, and Miyamoto *conducted experiments.* Nakamura and Fushimi *contributed new reagents.* Mandai, Sako, Kurimoto, Shimizu, Maeda, Miyamoto, and Kimura *performed data analysis.* Mandai and Kimura *wrote or contributed to the writing of the manuscript* *.*
