# Supplementary material for: T‐495, a novel low cooperative M1 receptor positive allosteric modulator, improves memory deficits associated with cholinergic dysfunction and is characterized by low gastrointestinal side effect risk
Source: Pharmacol Res Perspect. 2020 Jan 28;8(1):e00560. doi: 10.1002/prp2.560 (PMC6986443; doi:10.1002/prp2.560)
Supplement: Supplementary file 1 [file PRP2-8-e00560-s001.pdf]

T-495, a novel low cooperative M_1 receptor positive allosteric modulator, improves memory deficits associated with cholinergic dysfunction and is characterized by low gastrointestinal side effect risk

Takao Mandai[a], Yuu Sako[a], Emi Kurimoto[a], Yuji Shimizu[a, b], Minoru Nakamura[a], Makoto Fushimi[a], Ryouta Maeda[a], Maki Miyamoto[c], and Haruhide Kimura[a]

[a] Neuroscience Drug Discovery Unit, Research, Takeda Pharmaceutical Company Limited, 26-1, Muraoka-Higashi 2-chome, Fujisawa, Kanagawa 251-8555, Japan (T.M., Y.S., E.K., Y.S., M.N., M.F., R.M., and H.K.)

[b] Biomolecular Research Laboratories, Research, Takeda Pharmaceutical Company Limited, 26-1, Muraoka-Higashi 2-chome, Fujisawa, Kanagawa 251-8555, Japan (Y.S.)

[c] Drug Metabolism and Pharmacokinetics Research Laboratories, Research, Takeda Pharmaceutical Company Limited, 26-1, Muraoka-Higashi 2-chome, Fujisawa, Kanagawa 251-8555, Japan (M.M.)

Supplemental Table S1. in vitro selectivity of T-495 at 10 μM

Enzyme, receptor, or ion channel	% inhibition at 10 μM	Enzyme, receptor, or ion channel	% inhibition at 10 μM	Enzyme, receptor, or ion channel	% inhibition at 10 μM
ATPase, Ca^{2+}, Skeletal Muscle	0	Adrenergic β_2	15	Histamine H_1	2
ATPase, Na^+/K^+, Heart	4	Adrenergic β_3	7	Histamine H_2	22
Carbonic anhydrase II	-9	Androgen (testosterone)	3	Imidazoline I_2, central	76
Catechol-O-Methyl Transferase (COMT)	2	Angiotensin AT_1	23	Insulin	-10
Choline esterase, acetyl, ACES	-2	Angiotensin AT_2	0	IP (PGI2)	16
Cyclooxygenase-1 (COX-1)	-14	Bradykinin B_1	2	Melatonin MT_1	29
Cyclooxygenase-2 (COX-2)	31	Bradykinin B_2	-4	Muscarinic M_1	15
HMG-CoA reductase	-10	Calcium channel L-type, benzothiazepine	-5	Muscarinic M_2	8
Lipoxygenase (5-LO)	15	Calcium channel L-type, dihydropyridine	27	Muscarinic M_3	20
Monoamine oxidase A (MAO-A)	36	Calcium channel L-type, phenylalkylamine	19	Nicotinic acetylcholine	-4
Monoamine oxidase B (MAO-B)	6	Calcium channel N-type	0	Opiate δ_1 (OP1, DOP)	4
Nitric oxide synthase, inducible (iNOS)	0	Cannabinoid CB_1	14	Opiate κ (OP2, KOP)	14
Nitric oxide synthase, neuronal (nNOS)	5	Cannabinoid CB_2	12	Opiate μ (OP3, MOP)	12
Peptidase, factor Xa	-2	Cholecystokinin CCK_1 (CCK_A)	2	Potassium channel (K_{ATP})	2
Matrix metalloproteinase-1 (MMP-1)	5	Cholecystokinin CCK_2 (CCK_B)	10	Potassium channel (SK_{CA})	1
Matrix metalloproteinase-7 (MMP-7)	3	Dopamine D_1	-7	Progesterone PR-B	21
Matrix metalloproteinase-13 (MMP-13)	-26	Dopamine D_{2L}	4	Serotonin 5-HT_{1A}	8
Metalloproteinase, Neutral Endopeptidase	10	Dopamine D_{2S}	15	Serotonin 5-HT_{2A}	20
Phosphodiesterase PDE3	2	Dopamine D_3	-3	Serotonin 5-HT_{2B}	28
Phosphodiesterase PDE4	22	Dopamine $D_{4.2}$	3	Serotonin 5-HT_{2C}	10
Phosphodiesterase PDE4D2	2	Endothelin ET_A	-4	Serotonin 5-HT_3	-3
Phosphodiesterase PDE5	48	Estrogen Receptor (non-selective)	5	Serotonin 5-HT_4	22
Phosphodiesterase PDE6	6	$GABA_A$, chloride channel	45	Sigma, non-selective	25
Phosphodiesterase PDE10A2	5	$GABA_A$, flunitrazepam, central	12	Sodium channel, Site 2	1
Protein kinase C (PKC), non-selective	0	$GABA_A$, muscimol, central	-1	Tachykinin NK_1	-2
Protein kinase A (PKA)	-4	$GABA_B$, non-selective	4	Tachykinin NK_2	-20
Protein kinase, ROCK1	-22	$GABA_{B1A}$	5	Tachykinin NK_3	5
Protein tyrosine kinase, EGF receptor	9	$GABA_{B1B}$	6	Transporter, dopamine (DAT)	58
Steroid 5α-reductase	2	Glucocorticoid	25	Transporter, GABA	-7
Xanthine oxidase	7	Glutamate, AMPA	20	Transporter, norepinephrine (NET)	17
Adenosine A_1	15	Glutamate, kainate	13	Transporter, serotonin (SERT)	17
Adenosine A_{2A}	-4	Glutamate, NMDA	14	Transporter, vesicular monoamine (non-selective)	12
Adenosine A_{2B}	7	Glutamate, NMDA, glycine	15	Vasopressin V_{1A}	7
Adrenergic α_1, non-selective	11	Glutamate, NMDA, phencyclidine	3	Vasopressin V2	-7
Adrenergic α_2, non-selective	26	Glycine, strychnine-sensitive	9		
Adrenergic β_1	4	Growth hormone secretagogue (ghrelin)	8		

Supplemental Table S2. Concentrations of T-495 in rat plasma and brain

Dose (mg/kg, p.o.)	3	10	30
Plasma (ng/mL)	49.6 ± 14.5	298.0 ± 75.2	1045.5 ± 357.5
Brain (ng/g)	66.2 ± 18.5	304.1 ± 82.8	1005.0 ± 329.5
Kp	1.4 ± 0.1	1.0 ± 0.1	1.0 ± 0.1

Data are presented as the mean ± SD (n = 6). Concentration of T-495 in the hippocampus was regarded as the concentrations in the brain.

Supplemental Table S3. Concentrations of MK-7622 in rat plasma and brain

Dose (mg/kg, p.o.)	1	3	10
Plasma (ng/mL)	99.2 ± 27.4	217.9 ± 42.9	944.4 ± 246.3
Brain (ng/g)	14.5 ± 6.3	39.3 ± 5.6	220.4 ± 72.2
Kp	0.14 ± 0.04	0.19 ± 0.04	0.23 ± 0.02

Data are presented as the mean ± SD (n = 6). Concentration of MK-7622 in the hippocampus was regarded as the concentration in the brain.

Supplemental Table S4. Concentrations of T-495 and MK-7622 in the plasma and brain of wild-type and M_1R KO mice

	T-495 (10 mg/kg, p.o.)		MK-7622 (20 mg/kg, p.o.)	
	Wild-type	M_1R KO	Wild-type	M_1R KO
Plasma (ng/mL)	1689.9 ± 280.4	1593.4 ± 265.7	2235.1 ± 376.5	2194.6 ± 311.8
Brain (ng/g)	1026.5 ± 145.2	856.0 ± 78.4	582.1 ± 132.9	456.1 ± 85.8
Kp	0.62 ± 0.10	0.55 ± 0.09	0.26 ± 0.06	0.21 ± 0.03

Data are presented as the mean ± SD (n = 10). Concentrations of T-495 and MK-7622 in the hippocampus were regarded as the concentration in the brain.

Supplemental Table S5. Basal IP1 levels in the mouse hippocampus after repeated T-495 treatment for 13 days

Pretreatment	IP1 level (nmol/mg protein)
Vehicle	1.05 ± 0.04
T-495 (10 mg/kg, p.o.)	1.09 ± 0.03

Data are presented as the mean ± SEM (n = 10).

Supplemental Table S6. Basal IP1 levels in the mouse hippocampus after repeated MK-7622 treatment for 13 days

Pretreatment	IP1 level (nmol/mg protein)
Vehicle	0.93 ± 0.03
MK-7622 (10 mg/kg, p.o.)	0.99 ± 0.05

Data are presented as the mean ± SEM (n = 10).

Supplemental Table S7. Concentrations of T-495 in mouse plasma and brain after single and repeated treatment for 13 days

	Vehicle-pretreated		T-495 (10 mg/kg, p.o.)-pretreated	
	T-495 (10 mg/kg, p.o.)	T-495 (30 mg/kg, p.o.)	T-495 (10 mg/kg, p.o.)	T-495 (30 mg/kg, p.o.)
Plasma (ng/mL)	1148.2 ± 214.6	4894.9 ± 923.4	1299.8 ± 337.8	5422.1 ± 1745.5
Brain (ng/g)	955.7 ± 122.2	3654.0 ± 744.2	878.9 ± 158.8	4089.3 ± 599.2

Data are presented as the mean ± SD (n = 10). Concentration of T-495 in the hippocampus was regarded as the concentration in the brain.

Supplemental Table S8. Concentrations of MK-7622 in mouse plasma and brain after single and repeated treatment for 13 days

	Vehicle-pretreated		MK-7622 (10 mg/kg, p.o.)-pretreated	
	MK-7622 (10 mg/kg, p.o.)	MK-7622 (30 mg/kg, p.o.)	MK-7622 (10 mg/kg, p.o.)	MK-7622 (30 mg/kg, p.o.)
Plasma (ng/mL)	624.0 ± 61.4	2342.4 ± 517.7	489.9 ± 86.4	2430.4 ± 550.5
Brain (ng/g)	136.6 ± 23.1	787.0 ± 284.1	126.0 ± 28.7	813.1 ± 262.0

Data are presented as the mean ± SD (n = 10). Concentration of MK-7622 in the hippocampus was regarded as the concentration in the brain.

Supplemental Table S9. Side effects observed after oral administration of T-495 in combination with donepezil in rats

Group (dose, mg/kg, p.o.)	Loose, mucous stool, or diarrhea	Convulsion	Lacrimation	Salivation	Myosis	Fasciculation
Vehicle	0/6	0/6	0/6	0/6	0/6	0/6
Donepezil (0.1)	0/6	0/6	0/6	0/6	0/6	0/6
T-495 (0.3)	0/6	0/6	0/6	0/6	0/6	0/6
Donepezil (0.1) + T-495 (0.3)	0/6	0/6	0/6	0/6	0/6	0/6

T-495 (0.3 mg/kg, p.o.) was administered to rats 30 minutes before the administration of donepezil (0.1 mg/kg, p.o.). Observations were carried out at 10 and 30 minutes and 1, 2, 4, and 6 hours after the administration of donepezil. The data are presented as the ratio of rats exhibiting side effects to the total number of rats (n = 6).

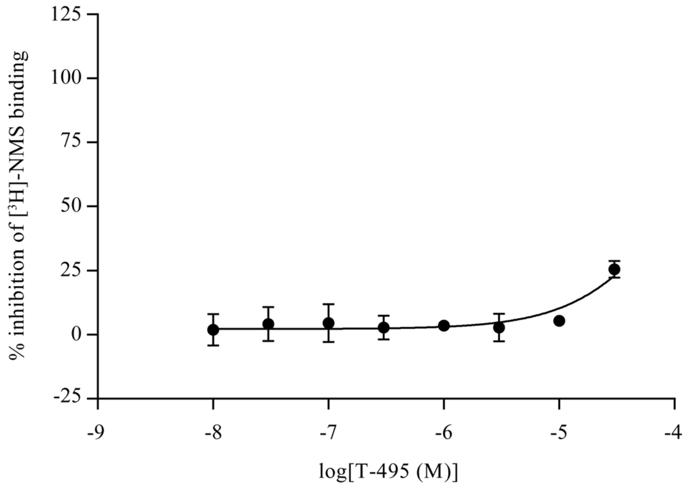

Supplemental Figure S1. Effects of T-495 on [^{3}H]NMS binding in cell membranes from human M$_1$R-expressing cells.

Nonspecific binding was defined in the presence of 10 µM atropine. Data are presented as the mean ± SD (n = 3).
